# Supplementary material for: Drug repurposing for aging research using model organisms
Source: Aging Cell. 2017 Jun 16;16(5):1006–15. doi: 10.1111/acel.12626 (PMC5595691; doi:10.1111/acel.12626)
Supplement: Supplementary file 7 — Data S1 Zip‐Archive of all report cards. [file ACEL-16-1006-s007.zip › RC_DMJ.pdf]

DMJ

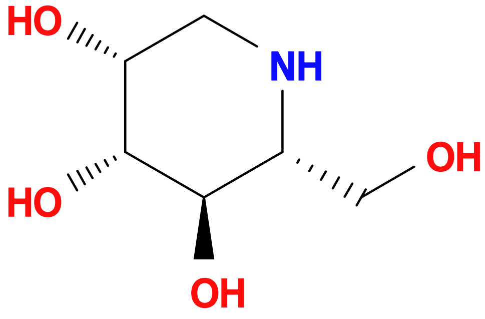

Database identifiers

ChEMBLCompound	CHEMBL84844
eMolecules	30512955

Ranking

	Rank	Score
Drosophila	380/697	0.382
C. elegans	354/591	0.166

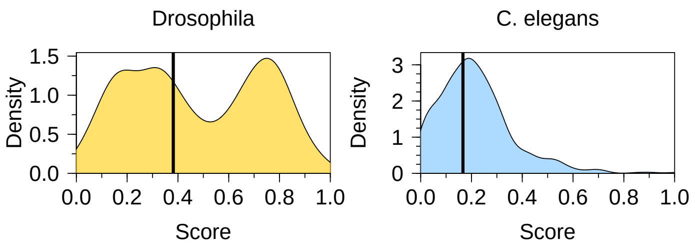

	Ageing implication	Domain conservation	Binding site conservation	Binding affinity	Bioavailability	Lipinski	Promiscuity	Purchasability	Drug approval	**Total**
Drosophila	1.0	0.704	1.0	0.445	(0.9)	0.0	-0.0	0.1	0.0	0.382
C. elegans	1.0	0.692	1.0	0.445	0.217	0.0	-0.0	0.1	0.0	0.166

Names

- (2R,3R,4R,5R)-2-(hydroxymethyl)piperidine-3,4,5-triol
- (2R,3R,4R,5R)-2-methylolpiperidine-3,4,5-triol
- 1,5-dideoxy-1,5-imino-d-mannitol
- 2-hydroxymethyl-piperidine-3,4,5-triol
- 3,4,5-Piperidinetriol, 2-(hydroxymethyl)-, (2R,3R,4R,5R)-
- BSPBio$_0$00991*Deoxymannojirimycin*
- Nsc625109 (hydrochloride)
- Ox5

Roles

ChEBI entry None has no roles

Status

Approved drug (according to ChEMBL)	No
Number of Rule of 5 violations	0
Binding affinity to original target in log units (RF-Score prediction)	4.78
Burns *C. elegans* bioavailability prediction	-4.62

Compound Target Characteristics

Endoplasmic reticulum mannosyl-oligosaccharide 1,2-alpha-mannosidase

Best gene implication in ageing for this target family came from gene P53625 annotated in UniProt release 2014_02. Annotation GO 8340 (determination of adult lifespan) was Inferred from Mutant Phenotype

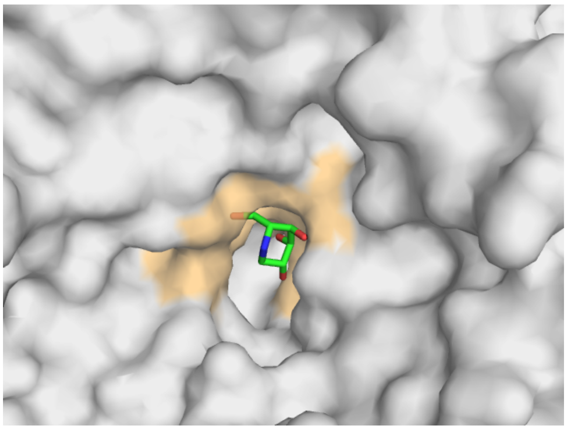

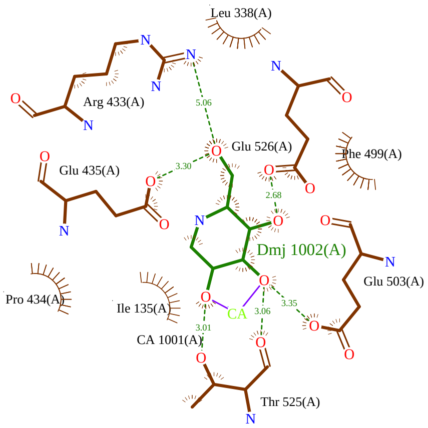

protein	amino acids contacts (binding site)
PDB:1g6i:chainA:P32906	I R L R P E F E T E
tr:A6NGN6:A6NGN6_HUMAN	I R L R P E F E T E
tr:B1AJZ5:B1AJZ5_HUMAN	I R L R P E F E T E
sp:Q9NR34:MA1C1_HUMAN	I R L R P E F E T E
sp:O60476:MA1A2_HUMAN	I R L R P E F E T E
tr:D3Z979:D3Z979_RAT	I R L R P E F E T E
tr:D3ZR49:D3ZR49_RAT	I R L R P E F E T E
tr:G3V9N9:G3V9N9_RAT	I R L R P E F E T E
tr:Q6NXK9:Q6NXK9_MOUSE	I R L R P E F E T E
sp:P39098:MA1A2_MOUSE	I R L R P E F E T E
sp:P45700:MA1A1_MOUSE	I R L R P E F E T E
tr:Q544T7:Q544T7_MOUSE	I R L R P E F E T E
sp:P53625:MA122_DROME	I R L R P E F E T E
sp:P53624:MA121_DROME	I R L R P E F E T E
tr:Q8IRL4:Q8IRL4_DROME	I R L R P E F E T E
tr:P90787:P90787_CAEEL	I R L R P E F E T E
sp:Q18788:MAN12_CAEEL	I R L R P E F E T E
sp:P32906:MNS1_YEAST	I R L R P E F E T E

protein	whole protein ident	simil	domain-based ident	simil	contact-based ident	simil
PDB:1g6i:chainA:P32906	1.0	1.0	1.0	1.0	1.0	1.0
tr:A6NGN6:A6NGN6_HUMAN	0.26	0.55	0.28	0.59	1.0	1.0
tr:B1AJZ5:B1AJZ5_HUMAN	0.28	0.62	0.3	0.66	1.0	1.0
sp:Q9NR34:MA1C1_HUMAN	0.23	0.54	0.31	0.68	1.0	1.0
sp:O60476:MA1A2_HUMAN	0.26	0.54	0.34	0.69	1.0	1.0
tr:D3Z979:D3Z979_RAT	0.24	0.54	0.31	0.68	1.0	1.0
tr:D3ZR49:D3ZR49_RAT	0.25	0.54	0.33	0.69	1.0	1.0
tr:G3V9N9:G3V9N9_RAT	0.25	0.53	0.33	0.69	1.0	1.0
tr:Q6NXK9:Q6NXK9_MOUSE	0.24	0.54	0.31	0.68	1.0	1.0
sp:P39098:MA1A2_MOUSE	0.26	0.54	0.34	0.7	1.0	1.0
sp:P45700:MA1A1_MOUSE	0.25	0.53	0.33	0.69	1.0	1.0
tr:Q544T7:Q544T7_MOUSE	0.25	0.53	0.33	0.69	1.0	1.0
sp:P53625:MA122_DROME	0.24	0.54	0.31	0.69	1.0	1.0
sp:P53624:MA121_DROME	0.23	0.53	0.31	0.69	1.0	1.0
tr:Q8IRL4:Q8IRL4_DROME	0.22	0.51	0.31	0.69	1.0	1.0
tr:P90787:P90787_CAEEL	0.23	0.58	0.27	0.66	1.0	1.0
sp:Q18788:MAN12_CAEEL	0.24	0.56	0.3	0.68	1.0	1.0
sp:P32906:MNS1_YEAST	1.0	1.0	1.0	1.0	1.0	1.0

alpha-Man-I (FBgn0259170) associated phenotypes

chemical sensitive, dominant, immune response defective, long lived

(Information from FlyBase)

alpha-Man-I (UniProt:P53625) annotation

Function: Involved in the maturation of Asn-linked oligosaccharides. Progressively trim alpha-1,2-linked mannose residues from Man(9)GlcNAc(2) to produce Man(5)GlcNAc(2).
Cofactor: Ca(2+)
Pathway: Protein modification; protein glycosylation.
Subcellular location: Golgi apparatus membrane; Single-pass type II membrane protein.
Tissue specificity: Complex spatial distribution during embryogenesis, including expression in lobula plate giant neurons. Also expressed in adult wing and eyes.
Developmental stage: Expressed both maternally and zygotically during embryonic stages.

(Information from UniProt)

alpha-Man-I (UniProt:P53624) annotation

Function: Involved in the maturation of Asn-linked oligosaccharides. Progressively trim alpha-1,2-linked mannose residues from Man(9)GlcNAc(2) to produce Man(5)GlcNAc(2).
Cofactor: Ca(2+)
Pathway: Protein modification; protein glycosylation.
Subcellular location: Golgi apparatus membrane; Single-pass type II membrane protein.
Tissue specificity: Complex spatial distribution during embryogenesis, including expression in lobula plate giant neurons. Also expressed in adult wing and eyes.
Developmental stage: Expressed both maternally and zygotically during embryonic stages.

(Information from UniProt)

C52E4.5 (UniProt:Q18788) annotation

Function: Involved in the maturation of Asn-linked oligosaccharides. Progressively trim alpha-1,2-linked mannose residues from Man(9)GlcNAc(2) to produce Man(5)GlcNAc(2) (By similarity).
Cofactor: Ca(2+)
Pathway: Protein modification; protein glycosylation.
Subcellular location: Membrane; Single-pass type II membrane protein.

(Information from UniProt)
